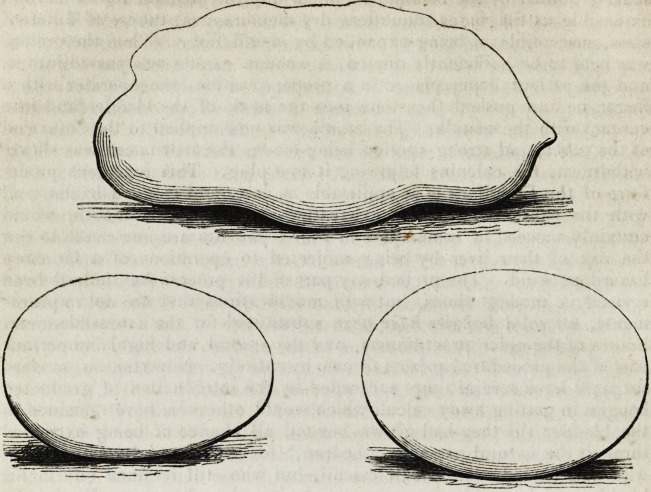# Nouvelles Considérations sur les Rétentions d'Urine, suivies d'un Traité sur les Calculs urinaires, sur la manière d'en connaitre la nature dans l'intérieure de la Vessie, et la possibilité d'en opérer la destruction sans l'opération de la Taille

**Published:** 1841-10

**Authors:** 


### ART. VI.

1. *Nouvelles Considérations sur les Rétentions d'Urine, suivies d'un Traité sur les Calculs urinaires, sur la manière d'en connaitre la nature dans l'intérieure de la Vessie, et la possibilité d'en operer la destruction sans l'opération de la Taille.* Par le Dr. CIVIALE.—*Paris*, 1823. 8vo, pp. 172.
2. *Exposé des diverses Procédés employés jusqu'à ce jour pour guérir de la Pierre sans avoir recours à l'opération de la Taille.* Par J. LEROY D'ETIOLLES, M.D.—*Paris*, 1825. 8vo, pp. 232.
3. *De la Lithotritie ou Broiement de la Pierre dans la Vessie.* Par le Dr. CIVIALE.—*Paris*, 1827. 8vo, pp. 254 and lx.
4. *Tableau historique de la Lithotritie.* Par J. LEROY D'ETIOLLES.—*Paris*, 1831. 8vo, pp. 102.
5. *Du Traitement Médicale des Calculs urinaires et particulièrement de leur Dissolution par les Eaux de Vichy.* Par CH. PETIT, M.D.—*Paris*, 1834. 8vo. pp. 58.
6. *Parallèle des divers Moyens de traiter les Calculeux.* Par le Dr. CIVIALE.—*Paris*, 1836. 8vo, pp. 492.
7. *Essai sur la Dissolution de la Gravelle et des Calculs de la Vessie.* Par A. CHEVALLIER.—*Paris*, 1837. 8vo, pp. 50.
8. *Nouvelles Observations de Guérisons de Calculs urinaires au moyen des Eaux thermales de Vichy.* Par CH. PETIT, M.D.—*Paris*, 1837. 8vo, pp. 102.
9. *Traité de l'Affection Calculeuse.* Par le Dr. CIVIALE.—*Paris*, 1838, 8vo, pp. 714.
10. *Exposé d'un Rapport fait à l'Académie de Médecine, relative à des Expériences sur l'efficacité des Eaux de Vichy contre la Pierre.* Par CH. PETIT, M.D.—*Paris*, 1839. 8vo, pp. 51.
11. *Histoire de la Lithotritie.* Par J. LEROY D'ETIOLLES.—*Paris*, 1839. 8vo, pp. 120.
12. *Du Traitement médical et préservatif de la Pierre et de la Gravelle.* Par le Docteur CIVIALE.—*Paris*, 1840. 8vo, pp. 452.
13. *A Treatise on Stricture of the Urethra, with an Appendix on Dilatation by Fluid Pressure, &c.* By JAMES ARNOTT, M.D. Second Edition.—*London*, 1840. 8vo, pp. 234.

THE number of capital surgical operations performed at the present day is generally held to be smaller than it was once; and it is quite certain that the general character of operative surgery has undergone a great change within even a limited period of time. The surgery of the middle ages was as barbarous and sanguinary as possible; the instruments employed were literally instruments of torture, and the chafing-dish and searing-iron were held as necessary to the surgeon then, as lint and sticking-plaster have become of late. The surgery of former times was cruel, then, and there is every reason to believe that operations were frequent. In the present day, on the contrary, surgery is as gentle as in the nature of things it can be made, and the glory now is to avoid an operation, not to have it to do. To us, however, there appears to have been one exception as regards the general disposition to operate, and this is in reference to Stone in the Bladder. Surgeons have never shown themselves solicitous about any means proposed for the relief of this dreadful malady that did not involve an operation; and we can almost pardon them for their anxiety to preserve lithotomy. Among the great operations, there is certainly none to compare in point of brilliancy and eclat with that by which a fellow-mortal is freed in a few moments from the tortures of stone: truly it is a temporal redemption that is achieved for the sufferer; and for our own part we cannot suppose that the leader of armies and of navies in the moment of victory experiences higher emotion than the skilful surgeon when he exhibits to his patient the cause of his long suffering, and tells him that now it is at an end! And is it not a most touching sight to see one man commit himself, bound hand and foot, voluntarily made incapable of resistance, into the hands of another for life and deliverance, or for death? and is it not an office of singular sacredness and responsibility which that other undertakes when he consents to receive this man into his care upon such conditions! Alas, that the courage displayed, that the responsibility incurred, should so often fail of the end proposed! for of all of every age who undergo the operation for stone, one in seven or eight will be lost; and of all who submit to it in the interval when man's life is most truly precious, when he is the mainspring in the varied business of the world, one at least in three or four will perish. No wonder then that, despite the benefits which the operation for stone has conferred, men not devoted to its practice have long eagerly sought either to escape the necessity for appealing to it altogether, or to find for it some substitute. To give an account of the attempts that have in modern times been made in this direction will be the business of the following pages.

The means by which a stone in the bladder has been sought to be removed are these:

i. By effecting its solution; and this has been attempted in two ways: *a*, by the agency of medicines administered by the mouth; *b*, by chemical agents thrown into the bladder.

ii. By removing it through the urethra entire; and this plan has been tried in two ways: *a*, with previous dilatation of the urethra; *b*, without dilatation or previous preparation.

iii. By grinding or breaking down the stone in the bladder, and so removing it piecemeal through the urethra, (Lithotrity.)

iv. By removing it rapidly and at once through an incision practised about the neck or fundus of the bladder; the parts which oppose resistance to the exit of the stone being dilated when it is small, torn when it is large, (Lithotomy.)

v. By removing it without tearing, or violence of any kind, through an incision into the urethra in the perineum, and slow dilatation of the membranous and prostatic portions of that canal, and of the neck of the bladder, (Lithectasy, Cystectasy.)

I. Removal of the Stone by Solution.

1. *By means of medicines administered by the mouth.* Until the discovery of the chemical composition of urinary calculi was made, none but blind and empirical attempts in this direction could be undertaken. In a science like medicine, however, empiricism has sometimes led to fortunate results; and in reference to the treatment of stone by internal medicines, which from their supposed virtues were called *lithontriptics,* it may be said that if less were known, nearly as much had been accomplished, before science shed any light upon the way, as has been achieved since she poured a noon-day splendour upon it. The use of the alkaline earth, lime, is as old as the time of Pliny, and the medicines of Stephens and her immediate successors, if less elegant in their forms, were essentially of the same nature and of the same potency as those that are in use at the present hour. Very different degrees of credit, however, have been attached to the powers of lithontriptics at different times. Like other specific remedies they were proposed as of universal efficacy, and having failed to do impossibilities they were then proclaimed as useless. But whilst many of the articles which were once regarded as lithontriptics have deservedly shared the fate of the generality of specifics for individual diseases, others have undeservedly been involved in the same neglect and oblivion.

In the nature of things there are certain articles which influence the renal function in a way that bears directly upon the disposition of its product, the urine, to deposit one or other of its concrescible elements, and which must necessarily, therefore, be of avail if directed aright in cases of calculus. The confidence once universally reposed in alkaline medicines in cases of stone could not, therefore, have been all misplaced. But an unlucky star seemed still to have dominated the proposal of every remedy for stone save in the way of operation. The advocacy of a Fourcroy may have forced the consideration of such means upon the attention of the medical profession for a time; but opposed by the most influential of that class of practitioners who are almost alone consulted in cases of calculus, they have soon sunk back into the shade. The contest, however, in regard to the value of alkaline lithontriptic medicines is by no means yet decided; it has been renewed of late years, and has hitherto been waged with nothing like defeat to their advocates. On the contrary, the most candid enquiry would rather lead to the conclusion that the extent to which alkaline medicines may prove beneficial in cases of calculus has been under-estimated rather than over-estimated.

The alkalis when first propounded as of sovereign efficacy in calculous complaints, were exhibited either in the state of subcarbonate or pure. This implies administration in a large quantity of fluid; and then the conditions were fulfilled which were necessary to the best effects of these medicines. The concurring testimony of all the best authorities of the times gives us assurance of the signal benefit that was often derived from the use of Stephens's medicines as well as those of her immediate successors. Stephens's remedy consisted especially of a mixture of calcined egg-shells and Castile soap, which was always washed down with copious draughts of some simple vegetable infusion or decoction. The lithontriptics of all the medical practitioners of the same period were of the same essential nature. The ingenious Dr. Whytt relied upon about an ounce of Castile soap and two or three pints of lime-water in the course of the twenty-four hours. Dr. Chittick had a tin vessel of the capacity of two quarts filled with weak veal-broth sent to his house every morning for medicamentation, and this quantity of diluent with some solution of potash added to it, was the dose which each of his patients took during the day. Now, provided the stomach did not rebel against a course of this kind, we are perfectly certain, as chemists and as physiologists, that it was calculated to act beneficially in a large proportion of cases of stone; in all cases, to wit, in which calculi of lithic acid, of the lithate of ammonia, and of the triple or mixed phosphates are contained in the bladder. There is, perhaps, no fact in therapeutics better established on the basis of experience than the influence of weak alkaline solutions upon the generality of urinary concretions. Qualities can readily be communicated to the urine by the use of alkaline medicines, that give it positive solvent or disintegrating powers, which though not of any great amount, are nevertheless quite decided, and have only to be maintained for a sufficient length of time to prove of signal efficacy.

Much good, then, was done during the period that alkaline medicines were administered in this way; some were freed from their calculi entirely, and many more escaped from a life of absolute torture to one of comparative ease; for the alkalis have this most admirable quality in addition to the one they possess as direct solvents of stone, that they allay the irritability of the living tissues with which the foreign body is in contact to such an extent that frequently its presence ceases to be perceived, and the person with a stone in his bladder comes at last to be in little worse plight than another who has nothing of the kind.

The progress of chemistry and pharmacy by and by led to the preparation and prescription of the carbonated instead of the caustic alkalis as heretofore; and, used in the old way with plentiful dilution, these are as good medicines as the others; but the alkaline bicarbonates especially are so mild that they soon came to be administered in no more water than was necessary to get down the dose; and then they certainly lost a considerable portion of their efficacy. At a subsequent period, a respectable chemist, experimenting under the auspices of a surgeon possessing a wide-spread reputation, announced that calculi of the lithic acid, the most common of all, were not acted on by mere saturated solutions of the bicarbonated alkalis, a proposition which in proclaiming a truth also involved an error; and so the confidence of the profession and of the public came to be entirely shaken in the powers of these medicines to benefit the sufferers from stone, who were, therefore, taught to cast aside every hope of relief, save in recurrence to the operation of lithotomy. It was in fact during the period that the medical treatment of calculus was pursued with the greatest zeal that the surgeon began to show himself the powerful rival of the physician in that walk of practice which has derangements of the uro-poietic system for its object. This took place especially from the improvements made in the operation for stone about the beginning and middle of the last century by the circumforaneous operators generally known under the names of Frêre Jacques and Frêre Come. The religious character of the former of these apostles of lithotomy, and the exaggerated estimates that were formed of the successes of both, contributed powerfully to direct public attention to the treatment of calculus by mechanical means, and to instal the surgeon as sole referee in cases of calculus, to the exclusion consequently of the physician; and the history of medical science since this change took place assures us that, whilst unremitting attention has been bestowed upon the improvement of the mechanical means of treating stone in the bladder, medical means have until very lately been more and more neglected. The physician, indeed, is now scarcely consulted, save at second hand, in regard to stone in the bladder. In ninety-nine cases in a hundred the surgeon has already been spoken to by the sufferer, and his mind is soon made up as to the procedure in such a case—the stone must be cut out, or it must be crushed; and circumstances not being very untoward, the cutting or crushing operation is forthwith undertaken. There is even something in the shape of an apology to be offered for the conduct so commonly pursued in such circumstances. The medical means we possess of attacking stone in the bladder are very slow in their effects; and all know how difficult it is to manage the generality of patients in cases where perseverance is indispensable, and where the result has to be looked forward to at a distant date. In the present time, too, the generality of practitioners are but indifferently acquainted with urinary pathology; the subject is one that is little studied, or they have little confidence in lithontriptic medicines. The surgeon, indeed, may be little or no better provided than the physician in the important article of pathological knowledge, but he has sounds, and catheters, and the lithontriptors and the knife at hand, and in these he has unbounded faith. The patient with a stone in his bladder, then, who consults the physician, is put into the best plight possible, and then comes in the surgeon, and in two or three minutes' time exhibits to him the cause of all his sufferings firmly held between the chaps of a pair of iron tongs. A triumph of this kind is not lightly to be foregone; and so long as mechanical means of removing stone in the bladder are held on the whole to be a tolerably safe means to this end, it never will be foregone. Neither can we expect that any others, necessarily of slow operation and of which the triumph must be altogether without eclat, will be anxiously sought after or sedulously employed if found by the operating surgeon. Nevertheless we shall see in the sequel that all the mechanical modes hitherto employed in removing a stone from the bladder are fraught with so much danger both present and prospective to the patient, that the interests of humanity and of true science alike command us to go on and strive to improve upon aught that has ever been done in the way of removing stone by other means than operation; and further, should the necessity for operating arrive, to enquire whether there be not better methods of proceeding than those in common use at present.

The chemists, especially of France, have mostly shown themselves warm advocates for the treatment of gravel and stone by medical means. Fourcroy and Vauquelin, in particular, raised their voices in their day in favour of the reasonableness of this practice; and it is to another able chemist of the same country, M. Darcet, that we are indebted for its revival in very recent times. In a short essay on the mineral waters of Vichy, published in one of the volumes of the Annales de Chimie for 1826, M. Darcet called the attention of professional men to the property which these waters have of rendering the urine of the drinker alkaline, and to the advantage that might be taken of this circumstance in the treatment of stone in the bladder. He found that from three to four glasses of this mineral water, which contains about fifteen grains of the bicarbonate of soda per glass taken in the course of twenty-four hours, sufficed to keep the urine permanently alkaline. M. Darcet remarked, further, that the urine of those who drank the Vichy water was singularly transparent, though the portion which was secreted during the night was often high coloured, and, contrary to wont, that it even continued limpid after putrefaction had made great progress. Dr. Charles Petit appears to have been the first, after M. Darcet, to investigate the effects of the Vichy water in the direction which that excellent chemist particularly indicated. In his little work "On the Medical Treatment of Urinary Calculi," (Paris, 1834,) Dr. Petit already adduced what it is impossible to regard as other than strong evidence in favour of the solvent powers of these waters, which might aptly be spoken of as a solution of bicarbonate of soda presented to us by the hand of nature; and in the "Additional Observations" (1837), and the "Appendix" to the Tract of M. Patissier "On the use of the Vichy Water in Gout" (1840) of the same writer, the subject is continued and other and more signal instances of success are adduced. It is agreed on all hands that fragments of urinary calculi of lithic acid and lithate of ammonia, and of the mixed and triple phosphates, are speedily reduced in size by solution and disintegration when exposed to the action of the Vichy water out of the body; and several persons in whose bladders the presence of calculi had been ascertained by searching, either got rid of them entirely or had them notably reduced in size, whilst all the symptoms of stone were subdued whilst using this water internally. Much about the same time (1836), M. Robiquet, in a paper read before the Royal Academy of Medicine of Paris, adduced several instances of the successful exhibition of the bicarbonate of potash aided by plentiful dilution in cases of stone in the bladder, patients finally passing the kernels of stones which formerly were too large to enter the neck of the bladder, and to get rid of which the operation had been proposed. The range of cases in which the alkaline carbonates were found to be useful was greatly extended by the experiments of these various enquirers, particularly of Dr. Petit and M. Chevallier. It had long been generally allowed that weak solutions of the vegetable and mineral alkalis in the state of carbonates exerted more or less of a solvent effect upon calculi of the lithic acid; but it was hardly suspected that these substances had fully as much power over concretions of the phosphates, not, however, in the way of solvents, but of disintegrators, the alkali seizing upon the animal matter, which is a principal bond of union in the great majority of urinary calculi, and the particles of earthy salt then separating and subsiding in the shape of an amorphous powder. M. Darcet in his experiments found that even so solid a substance as a *compact bone*, exposed for a length of time to a solution of bicarbonate of soda in distilled water, was finally completely disintegrated; a solution of gelatin, a gelatinate of soda, composed the supernatant liquor; the earthy matter, phosphate mixed with a little carbonate of lime, formed the powdery precipitate which lay at the bottom of the vessel. Dr. Petit even found that calculi of the mixed and triple phosphates, exposed to the action of the Vichy water, suffered in many instances a more rapid loss of weight than those of lithic acid or the lithates.

These interesting and remarkable results could hardly fail to attract the attention of the medical world at large, and particularly of that portion of it in the hands of which the practice in calculous complaints principally lay at this time. And as lithotrity had now reached its climax, having been received by the public at large with a degree of enthusiasm which in the domain of medical science is only accorded to success in surgical operations, the lithotritors or stone-grinders, like a community threatened by neighbours with an invasion that might involve the loss of goods and hearth and home, soon raised their voices against Dr. Petit in especial, and against all others, chemists, pharmacists, physicians, who had lately aided and abetted in this heresy of daring to hope for succour to patients affected with stone in the bladder otherwise than by the means of an operation—their own operation in particular. M. Civiale has shown himself very forward in this hostility to the medical treatment of calculus; but he has been joined in it most cordially by M. Leroy, his ancient enemy, his constant rival. After many articles in the journals of the day, which were met in counter-blasts with admirable temper and excellent taste by M. Chevallier and Dr. Petit, M. Civiale concentrated his strength first in the two works placed nearly last in our list, one published in the course of the year 1838 and the other in 1840. The first of these two works, and almost as much might be said of the last, is beyond question one of the most learned and elaborate productions which has fallen from the medical press of the present age. We have somewhere seen a summary of the number of references to authors and authorities, ancient and modern, and in almost every language spoken on the surface of the civilized earth, which this remarkable book contains; they amount, we think, to something like a couple of thousand. Some of M. Civiale's countrymen have not scrupled to say that there is but one man in France at the present moment who was competent to put forth such a work, a man not less distinguished by his vast erudition than by the facility with which he writes and his unwearied industry; and this is not M. Civiale but M. Jourdan. It is not our purpose to analyze these laborious and somewhat overlong productions, the first extending to upwards of 700 and the second to nearly 500 ample pages of close print in a very small type. Suffice it to say that the reader will find the entire subject of the formation, chemical and physical characters, causes and effects of calculus exhausted in the first, and in the second all idea of finding a remedy for stone, save in a mechanical operation, particularly lithotrity, utterly scouted; and those who have recently ventured to entertain hopes of affording relief in any other direction treated with very slender ceremony.

Messrs. Civiale and Leroy, then, however opposed upon other points, are very brothers in their hostility to the notion of ridding the bladder of a calculus by chemical means. M. Leroy has introduced his "Histoire de la Lithotritie" (1839), with certain reflections on the solution of stone in the bladder, rich in reasonings wherefore the alkaline carbonate, as we find it in the Vichy water especially, should not succeed in dissolving or disintegrating an urinary calculus, but very poor in facts to support these reasonings, which, indeed, being built upon false principles, are unsupportable. What we regret more than anything else here is to find the particulars of one of the two cases which M. Leroy quotes in aid of his views not given either completely or correctly. The case in question, then, is that of M. G., who went to Vichy in the month of June, where he remained for thirty-nine days drinking the water and bathing every day. During the whole of this time M. G.'s general health was excellent, and he was believed to be cured of his gravel. But this was not so, for soon after his return to Paris he had several small concretions extracted by M. Leroy. These concretions M. Leroy stated to have been composed of a mixture of carbonate and urate of lime, and this composition he adduced as proof positive that they had been formed under the influence of the alkaline waters of Vichy. But M. Guibourt, who made the analysis on being applied to by Dr. Petit, the unwearied and in all his statements unimpeached apostle of the Vichy springs, replied that what M. Leroy had stated, *n'était nullement exact*, was nowise according to fact, that what he had found in the said concretions was a mixture of *carbonate and phosphate of lime*, which is the usual composition of prostatic calculi. The patient was in fact known to be labouring under a severe affection of the prostate. M. Leroy also stated in reference to M. G., that calculi had been reproduced four times in the course of three years, and always by so much the more speedily as the patient drank the Vichy water in larger quantity. But the patient in question assured Dr. Petit in the most positive manner that *he had never drunk any Vichy water either at Vichy or at home* before the month of June last, the date at which he came to drink at the fountain head, (Exposé d'un Rapport fait à l'Académie Royale de Médecine au nom d'une Commission, &c., pp. 32 and 34, Paris, 1839.) The zeal of our dear brothers across the channel seems to carry them somewhat far. It is indeed extremely difficult for us to arrive at the truth on every point connected with this subject.

The subject of lithotrity, as we shall by and by see, has from first to last been surrounded by an all but impenetrable crust of falsification, which it is as distressing as it is difficult to break through. And here it is plain that the lithotritists show themselves no more scrupulous in reporting cases having reference to the solution and removal of calculus by alkaline medicines than they have been truthful in rendering an account of the successes and mischances of lithotrity. Probably one of the documents the most to be relied on in regard to the value of the alkaline bicarbonate, as it exists in the Vichy water, is the "Exposé" just quoted.

The general conclusions in the report alluded to are these: "1st, Urinary concretions are attacked by the urine when this has been rendered alkaline by the use of the Vichy water taken internally and in the way of bath; 2d, It has not been proved that urinary concretions of such a size as to constitute proper calculi have been entirely removed by these waters;* 3d, Such a removal is nowise impossible; there is even con-

* This judgment was given before the case of Denis B. Jacob had occurred, which will be found mentioned immediately.

siderable likelihood of its being accomplished; 4th, The question can only be decided by experiment; 5th, The experiment does not seem to present any danger. The committee therefore request the minister of the public works, &c. to accede to the demand of M. Petit," to the effect that he might have a certain number of patients affected with stone in the bladder confided to his care at Vichy with a view to decide the question as to the power or impotency of the natural alkaline waters in this disease. It is impossible not to see that the report of the committee is extremely guarded; under any other circumstances, in connexion with almost any other pathological state, the facts adduced would have been held adequate to authorize a far bolder and more favorable tone. One patient, M. de Longperrier, having suffered for two years with symptoms of stone in the bladder, and having had the operation proposed to him as his only remedy, goes to Vichy, and after drinking the water for nineteen days he passes a calculus, the size of a large lentil, "remarkable," say the committee, "by the disappearance of the superficial layers, which do not cover or inclose each other completely," and from this time forward he recovers. Another patient, M. Lorigandie, is found by searching to have "a stone of middling size" in his bladder. After the Vichy waters have been drunk for twenty days, pieces of calculus are repeatedly expelled, and the patient is completely restored and so remains. Unfortunately this patient was not sounded after his recovery. A third patient, aged fifty-two, having been sounded was ascertained to have several calculi, estimated about the size of hazel nuts, in his bladder. After having made use of an alkaline solution, during a month the patient passed eleven small concretions, weighing together no more than four grains. From this time all the symptoms ceased, and the bladder being searched anew no stone was discovered. M. Valerix is known to have a small stone in his bladder. Under the use of the Vichy water he passes first one and then another fragment of a calculus, and recovers. The bladder searched is found empty. M. Fournier has a voluminous stone with a rough surface in his bladder. Sounded by M. Leroy on the 9th August, 1838, the stone is declared to be of the size of a large walnut. Under the use of the Vichy waters all the symptoms of stone are gradually mitigated; large quantities of gravelly masses are passed, and the patient is at length so free from complaint that it is believed his stone must be all eliminated; but on sounding it is found with difficulty, and is allowed by M. Leroy to have decreased in size; to Dr. Petit the difference between the size of M. Fournier's calculus on the 3d of July and its size at the end of September seemed comparable to the difference between a hen's egg and a pea. The patient was and long continued perfectly well. In reference to this case the committee say: "If this case still leaves a doubt as to the entire disappearance of the calculus, no one surely will be found to doubt of the notable diminution of size which this large stone must have undergone." We are happy, through the politeness of Dr. Charles Petit, to whom we ventured to address ourselves for farther information in regard to this interesting case, to lay the sequel before our readers. M. Fournier, then, continued the alkaline medicine at home for some little time; but suffering nothing he soon gave up his medicine, and would not believe that he had any remains of a stone in his bladder. He enjoyed uninterrupted health for more than a year. In the month of

October last he was seized with fever of a bad type, to which he fell a victim. On opening the body after death the long-shaped nucleus of a stone, the size of a shelled almond, was discovered. It is impossible not to see this case as little less satisfactory than it would have been had the last fragments of the large concretion which once occupied the bladder been dissolved or discharged. Several other cases of equal interest might be cited, but we prefer giving one which is perfectly conclusive, and for which we are indebted to the politeness of Dr. Petit.

Denis B. Jacob, having a stone of considerable size in his bladder, was placed under the care of Dr. Petit, at Vichy, in the season of 1839. The patient proved refractory, and followed the treatment prescribed irregularly. Remanded in the season of 1840 ; his stone was previously seized in various directions by the committee of hospitals, Messrs. Civiale, Blandin, and Berard, and ascertained to be of the diameters of 13, 14, and 15 lines. The treatment by the Vichy waters, begun on the 23d of June, 1840, was pursued with great regularity to the middle of September, the patient taking from 12 to 25 glasses of the water and a bath daily, and in addition having a stream of the water sent through his bladder by means of a double-current catheter, once, twice, and even thrice a day for some considerable time. In the beginning of August the patient began passing fragments of his stone, and at the same time obtained relief from his sufferings. On the 18th of September the patient was sent back to Paris, and having been sounded on two different occasions by the several members of the committee, it was formally declared that there was no longer any stone in the bladder.

Two grand objections are constantly raised to the use of alkaline or any other kind of internal medicine in cases of calculus; first, that much valuable time is lost in their administration ; and second, that the alkalies especially, far from dissolving or disintegrating urinary calculi, tend rather to cause precipitation from the urine, or at most to change one diathesis into another. The first objection is taken obviously upon the presumed inefficacy of internal remedies generally in cases of stone. This is a presumption, however, which very certainly is nowise warranted by one item in our knowledge of the pathology of calculus. Stone in the bladder, indeed, is not one of those diseases the natural tendency of which is to get better; on the contrary, it is one that tends ever to get worse, and finally to destroy life, without the successful interference of art in one way or another. But there really seems to be no fact better established in therapeutics than this: that the symptoms of stone, those symptoms which directly bring the life of the sufferer into jeopardy, are in the majority of instances either entirely subdued or greatly alleviated by a course of the bicarbonated alkaline or Vichy water. The Committee of the Royal Academy of Medicine give a definitive judgment upon this point. "It cannot but be admitted as a general proposition," says the reporter, "that during the administration of the Vichy waters the health of calculous patients is ameliorated, and that the urinary passages undergo no changes from their action which could make the operation of lithotrity or lithotomy ulteriorly more hazardous." On the contrary, we venture to add, if they have a soothing and *healing* influence, to use a common phrase, which we maintain they have been satisfactorily proved to possess, is it not obvious that they must, as a matter of course, render either of these operations much safer than it would have been, undertaken upon a patient with his bladder in a state of active inflammation, and his system fevered and disjointed by pain? Doubtless, many cases will occur which are too far gone to be benefited by the Vichy or other kind of earthy water. But does lithotrity or lithotomy supply a remedy for every case of stone that presents itself in practice?

The second objection, that the alkalis, far from dissolving urinary concretions, tend rather to cause precipitation from the urine, and to change one calculous diathesis into another, is one that is now of ancient date, that originated in a groundless assumption, and that has been answered over and over again. Urinary calculi of lithic acid and the lithates, and of the mixed and triple phosphates are certainly dissolved and disintegrated by weak solutions of the fixed alkaline carbonates, which moreover have no power whatsoever to cause precipitation from the urine, or to alter the diathesis. The urine of the female whose case Dr. Bostock recorded,* when taking *two ounces and a half of subcarbonate of soda daily*, was pale and perfectly clear and limpid; and the urine of the drinkers of the Vichy water has been universally remarked for its transparency.

The medical treatment of stone in the bladder has assumed an entirely new aspect of late years, and the subject is not yet by any means exhausted. We have said nothing of the virtues of the biborate of soda, which nevertheless passes readily into the blood and finds an exit by the kidney, and acts very rapidly upon concretions of the lithic acid and the lithates. Neither have we spoken of the benzoic acid, one of the few substances of its class which is readily absorbed into the system, and like the alkalis, finds its way out again by the kidney. It is but yesterday since this acid, from its physiological affinities and its known capacity to combine with the uric acid, and to convert a most insoluble into an extremely soluble substance, was recommended in gout by Mr. Ure, as calculated to prevent the deposition and even to effect the removal of the tophaceous masses of urate of soda, which are so commonly seen about the joints in inveterate cases of that disease; and if the urobenzoic acid which is found in the urine when benzoic acid is exhibited by the mouth have the power of combining with the earthy phosphates, and of forming with them compound salts of easy solubility, as we have been assured it has by the ingenious surgeon just named, we are weaponed afresh and more effectually than ever againt urinary concretions.

2. *By chemical reagents thrown into the bladder.* This is a method of treating stone from which every addition recently made to our knowledge would lead us to anticipate great and decisive results. Nevertheless, it is one which has not attracted so much attention lately as the indirect mode of treatment through medicines administered by the mouth. More than a century ago our countryman, Dr. Hales, appears to have been in labour with the idea of dissolving the stone by way of injection. In the course of his experiments he found that a certain menstruum of which the active ingredient was subcarbonate or carbonate of soda attacked and dissolved urinary calculi with considerable vigour. This menstruum he also ascertained could be infused into the bladder of a living animal without injury, and he invented a double-current catheter

* Medico-Chirurgical Transactions, vol. v.

for its easy and effectual application; but he never tried its effect upon a stone in the bladder of man. Dr. Hales's views were, however, carried into effect about twenty years later in another and far more clumsy way by Dr. Rutherford, of Edinburgh, with the assistance of Mr. Butter, then a clinical clerk in the Royal Infirmary, and with a result powerfully calculated to arrest attention. The subject of Dr. Rutherford's experiment was a man forty years of age, who had suffered from all the ordinary symptoms of stone in the bladder for four years. On searching the bladder "a stone was distinctly felt, and it seemed to be a large one." But under the use of castile soap by the mouth, and injections of lime-water into the bladder, the symptoms of stone were first relieved; the stone, on searching, was found with difficulty, and reduced in size, and finally every indication of its presence having vanished, the sound failed to discover aught in the bladder, and the man went home well.* It is truly remarkable that so plain and so conclusive a narrative as that which Dr. Rutherford has left us in Mr. Butter's little book should not have borne fruit. Had we records of the example there set having been vainly followed in numerous instances we should be content to reconcile ourselves to the neglect into which the treatment of stone by the way of injection had fallen. The medical treatment properly so called has indeed been revived, and with what we cannot but hold good promise of many triumphs; but the treatment by injection has not yet found its Petit. Nevertheless, the two methods ought to be held as inseparably conjoined. Beneficial as each in the nature of things is calculated to prove by itself, the two in conjunction ought to accomplish, and will accomplish far more than either separately. In Dr. Rutherford's case they were combined; the soap by the mouth rendered the urine alkaline, and the lime-water injection, besides its own disintegrating powers upon the stone, by depriving the neutral carbonate of soda of its acid, exposed the calculus to the action of a weak solution of caustic soda. The treatment by injection, however, has not been entirely neglected; the object which Gruithuisen had in view when he contrived instruments to perforate stones in the bladder, was not their destruction in the way of grinding, but that he might open up a passage to their centre for suitable solvents.† In pursuit of the same end Gruithuisen revived or reinvented the double-current catheter of Hales, for passing a continuous stream of fluid through the bladder; but this ingenious man, like Hales, seems never to have tried the measure he advocated. It was, however, had recourse to by Messrs. Magendie and Amussat, in the case of an English gentleman under their care, but with only partial success. Sir Benjamin Brodie was more fortunate, and the solvent which he used, a weak solution of nitric acid in distilled water, was original. By means of injections of this composition two concretions of the mixed phosphates were finally so much reduced in size as to escape by the urethra. Dr. Ritter, of Cassel, also succeeded in removing a stone from the bladder of a gentleman aged forty, by injections of weak solutions of the caustic alkalis, aided by the same medicines taken by the mouth. Lithotomy was to have been performed in this case. On the thirtieth day the patient was completely relieved. The whole of the debris of the calculus collected

* Butter, a Method of Cure for Stone, chiefly by Injections. 12mo, Edinb., 1754.
† Willis, on Urinary Diseases, p. 335.

together weighed about one ounce and a quarter. The patient was seen by Dr. Ritter several years afterwards in perfect health, never having had any return of calculous complaint.* In this interesting case the means pursued were of the most active kind, and were perfectly adapted to the end proposed. The particulars of another remarkable case are narrated by M. Irvine, Professor of Anatomy, &c., Geneva,† and deserve to be referred to in this place. Under the influence of an injection of simple water into the bladder every day for several months, a large calculus, which must have been of the phosphatic kind, was so completely dissolved and disintegrated that at last it was reduced to a mere shell, and then broke down into a hundred pieces within the bladder. This case unfortunately terminated fatally; the patient, a female, seems to have suffered so much in her general health before Irvine saw her that no moment propitious to any kind of manual interference could ever be seized. It is worth while to state that the object of the practitioner in this instance was not to dissolve the stone, but to dilate the neck of the bladder and urethra to such an extent as to admit of its extraction.

On the subject of remedying calculus by the means of injections then, it seems clear that so much has already been done as to hold out every inducement to perseverance. In the nature of things, indeed, perseverance guided by the better knowledge we now possess of the agents that attack urinary calculi, and of the manner in which these operate, must of necessity be crowned with success in a certain proportion of cases.

The mode of treating stone in the bladder by injection, is in accordance with the ascertained laws of chemical affinity, which are just as determinate and as much to be relied on as those of gravitation. This treatment is therefore reasonable. Farther, it has been applied with complete success in more than one instance, and on the ground of experience we can say that it will surely succeed if fairly tried again.

### II. Removal of the entire Calculi through the Urethra.

1. *With previous dilatation of the canal.* The fact that persons after suffering from symptoms of stone in the bladder often obtained relief by the accidental escape of the concretion amidst a flood of urine, and that others who suffered in the same way constantly felt the stream of urine interrupted by the stone falling against the vesical orifice of the urethra, must have led, at an early period in the history of medicine, to the use of measures calculated to aid the escape of calculi. Had the canal of the urethra been but a little wider, the stone that became engaged in its vesical extremity would have passed through. The most simple and natural method of favouring the passage of calculi was to enlarge the urethra; and accordingly we find that one of what we must presume to have been a very early means of ridding the bladder of calculi other than by the knife, had dilatation of the urethra as its essential element. Prosper Alpinus informs us in his Medecina Ægyptiorum that he saw more than one Egyptian practitioner extract calculi the size of an olive, and, as he says, even of a small walnut, through the urethra pre-

* Chemische und Medicinisch-praktische Bemerkungen über menschliche Harnsteine, von Hofrath Ritter, in Hufeland's Journal. Band xxv. s. 119.

† Bulletin des Sciences Médicales de la Société Médicale d'Emulation, t. v. p. 400.

viously dilated by the insufflation of air and the introduction of certain extensible cartilaginous (doubtless dry membranous) tubes, of different sizes, susceptible of being expanded by insufflation. When the urethra was held to be sufficiently dilated, a wooden canula was passed into it, and the patient being placed in a proper position, the operator with a finger in ano pushed the stone into the neck of the bladder and into contact with the canula. The mouth was now applied to the outer end of the tubes, and strong suction being made, the instrument was slowly withdrawn, the calculus following it as a plug. This ingenious procedure of the Egyptians is as applicable as ever, and in good hands, and with the substitution of an exhausting syringe for the mouth, would certainly succeed in some cases in which patients are now made to run the risk of their lives by being subjected to operations of a far more hazardous kind. The preliminary part of the process has indeed been revived in modern times, but with modifications that are not improvements, for solid bougies have been substituted for the extensible membranes of the older practitioners, and the second and highly important part of the procedure has been thrown by entirely. Nevertheless, modern surgeons have several times succeeded by the introduction of graduated bougies in getting away calculi which would otherwise have remained in the bladder till they had grown beyond all chance of being extracted through the natural passages. Ledran,* for instance, advised a patient who had passed many small calculi, but who still retained one in his bladder which would not come away, to introduce bougies, and when he made water to lean forward that the stone might be directed towards the outlet. On the fifth day of the treatment the patient got rid of his calculus, which was of the size of a large pea. Baron Boyer† also succeeded in getting away four considerable calculi by following the same precedure. Sir Benjamin Brodie‡ in like manner, by the use of the full-sized bougie and diluents, was so fortunate as to relieve a patient of three considerable calculi, for the removal of which an experienced surgeon had recommended the operation of lithotomy.

But if less confidence have been given to dilatation of the urethra in the male than we think it deserves, and if this means have been called into requisition less frequently than it might have been, the case as regards the *female* is different. Such confidence have surgeons now in the dilatability of the female urethra, that the use of the knife appears to have been for some considerable time past completely laid aside in the treatment of calculus among women. When such masses as those, the outlines of which are here subjoined, will pass through the female urethra, there never can be any pretext for incising it. The largest of the three calculi here figured, actually passed spontaneously, literally forcing a passage for itself. But this knowledge, and the advantage that has been taken of it, are not of yesterday. The dilatability of the urethra was well known to the old French writers on lithotomy. Folet, in his "Traité de Lithotomie," published at Paris in 1682, says in express terms, "il n'est pas croyable combien l'urèthre se dilate tant aux hommes qu' aux femmes." Ledran never made any incision in removing calculi

* Consult. de Chirurgie, p. 471. † Traité des Maladies Chirurg., t. ix, p. 308.

‡ On Diseases of the Urinary Organs.

from the female bladder. Bromfield* dilated the female urethra for the extraction of calculi with the bowel of a small animal inflated, &c. But these facts, and the practice connected with them, had fallen into oblivion, when they were again brought under the notice of the profession by Mr. Thomas† about thirty years ago, he having succeeded in removing an ivory implement three inches in length, which had been introduced into the bladder, after the use of no more than a couple of sponge tents of very moderate dimensions. These he found had so far dilated the urethra in about twelve hours, that he was enabled easily to pass his finger into the bladder and dislodge the intruder. The example once set was in this instance speedily followed, and the practice had soon many signal triumphs to boast. The only objection that can be urged to the practice as at present pursued is, that the instruments commonly employed to procure the dilatation are not the best that have been contrived, and that the process is all but invariably carried on too rapidly.

2. *Without previous dilatation of the urethra.* It had been occasionally observed, that catheters introduced into the bladders of persons who had suffered from retention of urine in consequence of the presence of gravel or calculus, were withdrawn with a small concretion sticking in their eyes; and this circumstance deserves attention, as having led to very important consequences. The accident alluded to must have occurred ever since canuli for the urethra or catheters were invented; but the first who attempted to take advantage of it was M. Bourguenod. In a modest paper in the 6th volume of the Annales de Montpellier, this practitioner gives the particulars of three cases, in which as many as five

* Chirurg. Observ. Lond. 1773. † Med. Chirurg. Trans. vol. i. Lond. 1812.

small calculi were successively removed from the bladder by its means. He used what the French call *sondes à demeure*—elastic catheters left permanently in the bladder. The eyes of the catheter were split to a certain extent, and the cessation of the flow of urine through the instrument was the signal that the concretion had entered the trap set for it. This generally took place in the course of three or four days. The concretion removed in one of the instances was as long and as thick as the end of the little finger; and in another the operation of lithotomy was rendered unnecessary by the successful issue of the simple expedient employed. The Baron Boyer* also informs us that upon a certain occasion on removing an elastic catheter he found a calculus of the size of a haricot bean sticking in one of the eyes of the instrument. The same occurrence has happened in the practice of many other surgeons. Mr. George Bell† has given the particulars of a case in which the accident furnished the patient himself with the hint of a method by which he succeeded in removing upwards of 150 small calculi, by means of an ample silver catheter used when the bladder was fully distended; and who derived so much relief from getting rid of all smaller concretions, that although one larger calculus still remained behind, he could never be brought to take the chances of lithotomy. Finally, the late—alas the late! Sir Astley Cooper, having left a catheter in the bladder of a patient affected with calculi of small size, on being informed that concretions had several times been found sticking in the eyes of the instrument, desired that he might be allowed to remove it himself next day; and this being done, a small stone was actually found impacted in one of its eyes. The advantage that might be taken of this circumstance immediately presented itself to the mind of the great surgeon: he resolved by means of a pair of delicate forceps to seize the calculi where they lay, and to extract them through the urethra. A suitable instrument was soon constructed with the assistance of the ingenious surgeon's-instrument-maker Weiss, and this was plied so successfully at intervals during several weeks, that the quarry became exhausted, and the patient went home well. The same method was shortly after resorted to in other instances, and with the like success.

This method of extracting stones from the bladder is however no new discovery; passing by the more ancient writers, it is particularly mentioned by Sanctorius,‡ and an instrument is described and figured which he contrived for this especial purpose. An instrument on the same principle, having two blades (Sanctorius's had three) was invented about a century ago by Dr. Hales, but this was rather for the extraction of stones arrested in the urethra. The same implement was reproduced by Mr. Hunter; and he or some contemporary (though we have failed to light on the passage) must have proposed going into the cavity of the bladder, and thence extracting calculi of smaller sizes, for the proposition may be found commented on in terms of great severity by M. Deschamps in his classical work, the "Traité pratique et dogmatique de la Taille," (4 vols. 8vo, Paris, 1796), which is in everybody's hands, and where a figure of Mr. Hunter's forceps is given. We have farther been favoured with an original drawing of a three-bladed forceps acting by its elasticity and the pressure of a canula,

* Op. cit. t. ix. p. 318. † Edinb. Journ. of Medical Science, vol. i.

‡ Comment. in Avicenn. fol. Venet. 1626.

which was repeatedly used by the late Sir William Blizard, at the London Hospital half a century ago, for the very purpose in question.

With regard to the value of the idea of extracting calculi from the bladder in the manner and by the means indicated, this has been very differently estimated by different writers. One excellent surgeon has proclaimed it as among the greatest achievements of modern surgery (Brodie); another has said that it could hardly fail to prove highly injurious (Syme); and a third has declared it imprudent and murderous (Deschamps). There must be cases, we believe, in which, in the absence of other and better means, this procedure would be found useful if cautiously instituted. But that the extraction of calculi by means of forceps through the unprepared urethra is an operation not without danger is quite certain; it is no such unmingled good as it was proclaimed and believed to be after Cooper's operation. Proof of this may be had by turning to a late volume of the London Medical Gazette, where a case is reported in which death ensued, though the forceps were in very competent hands, and no more than two attempts to use them were made.

### III. REMOVAL OF THE STONE PIECEMEAL, BY DRILLING, GRINDING, OR CRUSHING IT WITHIN THE CAVITY OF THE BLADDER.—LITHOTRITY.

The modern history of LITHOTRITY, by which is now understood destruction of the stone within the bladder by mechanical means of any kind, begins with the accounts we have of the case of Major-General Martin, the fullest and best of which are from his own pen. This distinguished officer was a native of Lyons, and arrived in India in the capacity of a common soldier; but from this humble position he soon emerged, and finally attained the very highest rank that could be won in the Honorable Company's military service, owing his advancement to his abilities and worth alone.* The first published account which we have seen of Gen. Martin's case is in the 1st volume of the Medical and Physical Journal (Lond. 1799). There is, however, a communication, earlier in point of date, though later in regard to the time of its publication, extant in the 10th volume of the Annales de Montpellier (Montpel. 1809). It is entitled "Fragment d'un lettre de M. Claude Martin, &c. à M. Pictet de Genève, communiqué par M. P. Fine," and bears date, Lucknow, Dec. 10th, 178(9?)5. The letter is in French, and is extremely interesting. The writer informs his friend, M. Pictet, that he had been so fortunate as to cure himself of a stone in the bladder, which he imagines must have been of large size, by means of a contrivance of his own, a file, curved to correspond with the canal of the urethra, and toothed at the end, so as to cut in the withdrawing. By standing up and

* On the tomb which he constructed for himself during his lifetime, on the underground floor of a "grotesquely magnificent house which he built at Lucknow," are these words from his own pen:

"MAJOR-GENERAL CLAUDE MARTIN,
BORN AT LYONS, JANUARY, 1738,
ARRIVED IN INDIA AS A COMMON SOLDIER,
AND DIED AT LUCKNOW [15TH OF SEPTEMBER, 1800],
PRAY FOR HIS SOUL!"

(*Emma Roberts, Scenes and Characteristics of Hindostan,* vol. ii.)

leaning forward the stone is brought to the neck of the bladder, and it is then that it must be attacked with the file. General Martin continued his operations through a succession of months, filing on an average three times a day, but often as many as ten and twelve times. "I filed to such purpose," he concludes, "that at length I brought away the last fragment of my stone, and am now perfectly well." General Martin's case has been noticed repeatedly since, more particularly in the 1st volume of the Journal of the Royal Institution in 1816, and by Dr. Marcet the year after, in his work on Calculous Diseases. But it remained without results long after its publication.

The next stage in the history of lithrotrity is unquestionably the publication of Dr. P. E. Gruithuisen's paper in the Medico-Chirurgical Journal of Salzburg in the year 1813. The title of this paper is as follows: *Are we then to abandon the long-cherished hope of removing stone in the bladder by mechanical or chemical means?* (Ob man die alte Hoffnung aufgeben sollte den Stein aus der Blase auf mechanische oder chemische Weise einst noch wegschaffen zu können?) and is truly a remarkable production. The author declares himself a strenuous advocate for the idea of dissolving the stone. He instituted experiments to show the extent to which calculi were attacked by being placed in a current of water, pure or holding different acids and saline substances in solution; and among other means he describes and figures a canula, through which was passed a noose of wire to secure the stone, in the same way as a loose cork is secured in a bottle, and a small trepan with which the concretion was to be perforated, its nature ascertained, and its very centre opened up to the action of the appropriate solvent. This brilliant idea was, however, never carried into practice; Gruithuisen's paper attracted no notice in the world of Germany, and remained unknown to the rest of Europe. It therefore influenced in no way the measures that were subsequently taken towards effecting the mechanical destruction of the stone in the bladder. The stream of improvement, in fact, did not set from Germany, but from England, where alone of all the countries of Europe was the subject of urinary pathology cultivated with anything like zeal or success about and previously to this time. In both France and Germany indeed the uropoietic system and its derangements seemed to have been long at a discount. With the discovery of the urate of ammonia calculus by Fourcroy and Vauquelin in 1799, the successful researches of French chemists in the domain of urinary concretions may be said to have begun and ended. What may be called the general and special medical "urinary pathology," too, found tongues and pens in England alone, to proclaim its importance, and to push it forward, about the end of the last and during the first quarter of the present century. France has no such names as those of Rollo, Cruikshank, Wells, Bostock, Marcet, Prout, Blackall, and Bright, in connexion with this important matter. Neither in reference to the operation for stone did she produce aught after the compilation of Deschamps (1796) that either added to knowledge or influenced practice; whereas in England, we observe the whole profession, as it were, for a long series of years labouring heart and hand at the improvement of lithotomy. The same may be said in regard to the diseases of the urethra: from the appearance of Chopart's work in 1792, nothing was contributed by our neighbours of any consequence till the year 1822, when M. Ducamp's treatise appeared, and this seems to have been borrowed in every essential particular from the English, more especially from Dr. James Arnott's book upon Strictures of the Urethra, which was published in the course of 1819. We shall by and by show what an important influence we imagine M. Ducamp's acquaintance with this book, and with English medical literature, had upon the invention of lithotrity. France, in short, had little or no share in all that led the way to what is now generally esteemed her highest surgical glory—the fashion of treating stone in the bladder by grinding and crushing. Returning to which subject we remark that

The third direct stage in the history of this invention in these times was the appearance of a paper entitled, Description of an Instrument for Destroying Urinary Calculi within the Bladder, with a plate; by John Elderton, surgeon, in the April number of the Edinb. Med. and Surg. Journal for 1819. This, in fact, is the first broad annunciation we possess of the principle of lithotrity. Mr. Elderton's instrument is ingenious, and under favorable circumstances would almost certainly have been used with success; the first softish stone to which it had chanced to be applied would have given way and crumbled into fragments between its blades. Unfortunately Mr. Elderton published his invention before he had proved its applicability; it was passed by unheeded as a vain speculation, and seems to have had no immediate influence on the treatment of stone in the bladder. Nevertheless, there is the honour due to Mr. Elderton of having the first invented what we hold to be an available instrument for the express purpose of destroying a stone in the bladder.

The fourth and most important step of all in the history of lithotrity, as we conceive, was the publication of "A Treatise of Stricture of the Urethra, by James Arnott, Lond. 1819," and of "Cases illustrative of the Treatment of Obstructions in the Urethra, Lond. 1821." In these works, however, allusion is only made incidentally to a mode of ascertaining the nature of a calculus still contained in the bladder. "If the smallest particle of the stone could be procured," says Dr. J. Arnott, (Cases, &c. p. 85), its chemical composition might with certainty be determined. With this view the following means may be adopted: When the stone comes to the orifice of the bladder, let an open pointed catheter (having of course a ball-ended wire filling it during the introduction) be passed till it touch the stone; by this a small circular saw like a trephine may then be introduced, to grate off from the calculus, by a few turns, a sufficient quantity of dust for examination." Dr. J. Arnott was at this time intent, in concert with his distinguished brother Dr. Neil Arnott, upon finding a means of destroying calculus in the bladder by solution. After indicating the method, by the injection of certain menstrua into the bladder, which had already been essayed, and speaking of the double current catheter which they had rediscovered for themselves, as affording important advantages over the old plan of proceeding, Dr. J. Arnott goes on to describe a bag of gilded cloth which had been contrived by his brother Dr. Neil Arnott, capable of being introduced into the bladder closed, but of being expanded there, and with which he thought the stone might be caught. This done, and the mouth of his bag closed again, he proposed to throw in strong solutions of acids or of alkalies, according to the ascertained nature of the stone, so as rapidly to destroy it. "The experiment of removing calculus," says Dr. J. Arnott, "was made by my brother upon a stone in a narrow-mouthed glass vessel with such an apparatus, and perfectly succeeded." Dr. J. Arnott goes on to speak of "the removal of the stone by mechanical attrition," and instances Col. Martin as having "thirty years ago removed a stone from his bladder by files introduced through the urethra." He also intimates that Dr. Darwin suggested or described an apparatus which might be used to break the stone into distinct portions. In spite of what he has already said of a means of ascertaining the nature of the stone, and in the very next sentence to that just quoted hinting that "better instruments might be constructed than any which have as yet been proposed for the purpose (of breaking up the stone)," our author nevertheless expresses himself as adverse to the idea, and even goes so far as to say that he thinks it scarcely probable the thing will ever be attempted.

But we have not yet done with Dr. James Arnott. In commenting upon the fact that stones of considerable size had often been expelled through the female urethra especially, either by the efforts of nature alone, or assisted by dilatation of the passage (op. cit. p. 111), he speaks of the Arabian mode of extracting calculi from the male bladder by dilating the whole length of the urethra, and by and by gives the particulars of a case in which a stone was removed from the bladder of a patient through an opening in the perineum after dilatation of the membranous part of the urethra and neck of the bladder by means of a new dilator. The subject of this case was a gentleman beyond the middle age, who had some nine months previously undergone the usual operation for stone, in the course of which the rectum had been wounded, so that a fistulous opening remained between the bladder and the bowel. In this state, and still suffering much from pain and irritability of bladder, the patient placed himself under the joint care of Drs. Neil and James Arnott, and Sir Astley Cooper. With a view to cure the fistula, Sir Astley Cooper made an opening into the urethra from the perineum, by which he passed a female catheter into the bladder, and immediately struck a stone. As it was likely to be small, Sir Astley did not object to the proposal now made by the Drs. Arnott, to try the effect of the new dilator in opening the passage for its removal. This instrument was accordingly used; and in the course of thirty hours the passage in the perineum, the membranous portion of the urethra, and the neck of the bladder, were opened up till they were about two inches and a quarter in circumference, or three quarters of an inch in diameter. The lithotomy forceps was then introduced by Sir Astley Cooper into the bladder, and the stone extracted. It was as large as a middling sized walnut. The operation was eminently successful. In four days the patient was able to retain his urine, and left his bed-chamber. On the ninth day the wound in the perineum was whole, and he began to take exercise abroad. This case unquestionably exerted a very considerable influence upon the views of practitioners at the time in this country. At the meeting of the Medico-Chirurgical Society, which was held on the 22d of June, 1819, it was particularly mentioned by Sir Astley Cooper, who spoke in high terms of the plan of operation pursued; and its publication immediately preceded Mr. Earle's paper on the means of breaking down large calculi, and Sir Astley Cooper's communication on the extraction of calculi from the male bladder without cutting. The knowledge of this remarkable case was, further, much more extensively spread by being given at length in the immediately ensuing number of Dr. James Johnson's Journal, in connexion with a very flattering review of Dr. Arnott's book on Stricture, which is highly lauded, and spoken of as the best in the English language on the subject. At the end of this very number of Dr. Johnson's publication, page 331, it is not unimportant to observe the name of M. Ducamp, Docteur en Médecine, rue St. Martin à Paris, among the list of recent subscribers. All that was passing in the medical world of England must consequently have been perfectly well known to Dr. Ducamp, who, indeed, had already signalized himself as an English scholar by translating, among other works, Bree's book on Asthma into French ; we have, besides, seen his name among the list of contributors in the department of English literature to a Parisian medical periodical publication of the time—the *Revue Médicale,* if we remember rightly.

In the course of our researches for information upon the various particulars embraced in this summary, we were struck by the essential similarity of the ideas and means of cure proposed by Arnott in his Treatise on Stricture (London, 1819), and Ducamp in his Traité des Rétentions d'Urine (Paris, 1822). It was obvious at once that Ducamp had made very free use of Arnott, though without the slightest acknowledgment, for he only quotes the English writer once (p. 171), and that is to accuse him of filching from Desault! This single citation of Arnott suffices of course to prove that the "Treatise on Stricture" was known to Ducamp, whose references besides to Home, Whateley, and Bell show him to have been familiar with the English works of highest authority on strictures of the urethra. It is not likely that he would have neglected the last publication on the subject, especially as he must have seen it spoken of in his copy of Dr. Johnson's Journal, as "the best that had appeared upon it in the English language." That Ducamp was himself guilty throughout his entire treatise of the sin with which he wrongfully charged Arnott in a single point there can be no doubt.

In the course of our farther researches for information on lithotrity in its embryo state, we were a good deal struck by observing Ducamp's name frequently quoted in the work which M. Leroy published in the year 1825, entitled "Exposé des diverses procédés employés jusque à ce jour pour guérir de la pierre sans avoir recours à l'opération de la Taille." The first implement which this gentleman imagined for perforating the stone was, he tells us, (Op. cit. p. 126,) "a button covered with asperities or a small trepan, supported upon a slender and flexible stem similar to that which supports the port-caustic of *Ducamp.*" He mentions Ducamp again at page 144 as having given him the idea of the bow to work his drill instead of the crank-handle, which was his own invention. He speaks of Ducamp a third time at page 151, and recommends an instrument similar to one which Ducamp had imagined for measuring the extent of strictures, as calculated to bring back the stone when tending to escape from the lithoprione or stone-saw. Leroy in the same work further describes and figures a pouch for catching the stone in the bladder, and at page 168 he says, "if this pouch be intended to receive solvent injections," &c. &c. Now it struck us as extraordinary on perusing all this that the man who had vindicated his claims to the character of ingenuity in one department of the diseases of the urinary organs, and who could give so many useful hints to his "*friends,*" should in another and still more interesting division of the same diseases have done nothing for himself. Our astonishment was increased when we saw that M. Leroy's ideas, so naïvely laid before us as his own, were essentially the same as those of Arnott; we had the trepan for instance for piercing the stone, the object in doing which was among other things to ascertain its nature, and we had even the pouch for catching the stone previously to dissolving it by chemical reagents. But our amazement at this harmony of views and means subsided in a very considerable degree when we chanced to turn to a work entitled "Recueil d'observations médicales confirmant la doctrine de Ducamp sur la cauterization de l'uréthre," &c., published by M. P. L. A. Nicod at Paris in 1825. Ducamp, who died prematurely and much regretted in the spring of 1823, seems to have pointed to Nicod as a fit successor in his practice, and M. Nicod in the preface to his work (Recueil, &c. p. xvi. et seq.) gives the following information, which we cannot help viewing as strongly confirmatory of our persuasion that the entire subject of lithotrity in France took its origin from Ducamp, who in his turn derived his ideas from the literature of England, particularly from the two publications of Dr. James Arnott. "To an inventive genius," says M. Nicod of his friend, "great dexterity, and rare powers of observation, Ducamp added an activity of mind that would have led him to other discoveries of equal importance, had he not been prematurely snatched away from science. His researches on the urinary passages had given him *the idea of an instrument intended to destroy calculi in the bladder without recurring to the operation of lithotomy*. . . . This instrument served subsequently as the model of that of M. Leroy, in taking advantage of which M. Civiale showed such address. Ducamp would probably have perfected his stone-breaker had he not hampered himself with the idea of a pouch in which he proposed to retain the fragments with a view to dissolve or to extract them subsequently." There is the stamp of truth upon this narration, the rather as these ideas of Ducamp are the same in effect as those of Arnott; he made a pouch in which he meant to catch the stone, and *having broken it into pieces* (and here he went beyond Arnott) to dissolve or wash it out. M. Leroy has hitherto maintained a character among his countrymen for candour and fairness. He has shown much of these excellent qualities to his rivals, doubtless; but them we opine he has always had upon the hip, to them he could afford to be generous; why has he carefully avoided the mention of his deceased friend Ducamp, in each and all of his publications as having invented an instrument for breaking stones in the bladder? He must have known that Ducamp had contrived something of the kind; Ducamp gave him the flexible stem upon which his piercer was supported, and made fit to act through a bent tube; Ducamp gave him the bow instead of the crank. Did not Ducamp do more? It were, perhaps, too much to put M. Leroy in foro conscientiæ on this occasion, but we cannot help expressing the wish that he would tell us what share Ducamp had in setting him to work at the destruction of the stone in the bladder, and what part the same ingenious man had in contriving the instruments originally constructed for this end. The circumstances attending the announcement of lithotrity in the year 1822 point with something like a necessity, indeed, to a common centre whence the ideas emanated; for no fewer than three competitors for the honour of having discovered lithotrity actually stepped upon the stage in France nearly at one and the same moment.

These competitors were Messrs. Amussat, Leroy, and Civiale. The first in point of time is M. Amussat. This gentleman had already been engaged in studying the structure of the urethra, and had called attention to the neglected fact that straight sounds are readily enough passed into the male bladder. The title of his communication in reference to lithotrity is remarkable. It is as follows: "Note sur la possibilité de sonder l'urèthre de l'homme avec une sonde tout à fait droite, &c., ce qui a donné l'idée d'extraire des petits calculs urinaires encore contenus dans la vessie et de briser les gros avec la pince de Hunter modifié," in Nouveau Journal de Médecine, Avril, 1822. This announcement is very striking. If there were an enquirer in the direction he took independent of his contemporaries, it was M. Amussat. He informs us that on the dead body and in presence of his pupils he had repeatedly practised the extraction of calculi from the bladder, and broken others of the size of walnuts within it, having none larger at his disposal. The instrument was composed of a couple of strong blades well toothed towards their extremities, opening by their elasticity through a canula. The stone being felt was firmly grasped between these blades by their being drawn within the canula, and the pressure thus exerted upon it broke it in pieces. But there was nothing of novelty in the ideas here. Mr. Hunter's forceps was already before the world as an instrument fitted to penetrate into the bladder and thence to extract calculi; and this had already been done in England not upon the dead body but in the living subject by Sir Astley Cooper, when he freed the bladder of Mr. Bullen of its eighty-four calculi without cutting. And with regard to the second idea, that, to wit, of breaking calculi which were too large to pass, the only new point was the strength of M. Amussat's instrument; for Mr. Weiss the cutler, informed of the difficulty or impossibility of removing calculi from the bladder that were above a certain and very moderate size with the forceps which he had already constructed, made another in the course of the same year (1821) upon a similar principle but of tempered steel, and of such strength that its power of resilience sufficed to crush calculi of considerable dimensions, especially when they happened to be soft.

The next in sequence is M. Leroy, the title of whose communication is short and to the point: "Note sur un nouveau procédé pour detruire la Pierre dans la Vessie, par M. James Leroy," in the Revue Médicale for June, 1822. In this note an instrument for seizing the stone is described. It consisted of a double or outer and inner canula of silver, eight inches long, between which a number, four or more, of watch-springs connected together by a button, which served to close the canula and admit of its introduction, were passed. These straight springs could be expanded by being pushed forward one after another, when they formed a kind of cage within which the stone being caught was readily fixed, and then perforated by means of a small circular saw or trepan passed down to it through the inner canula. The power of this instrument to reduce and remove piecemeal within the crown of the trepan those calculi that were too large to pass by the urethra is spoken of. It is farther stated that "the instrument may supply us with a means of turning the discoveries of modern chemistry to profit. . . . Among the reagents capable of dissolving a stone, there are several," it is observed, "that may be introduced into the bladder without detriment or danger; but in ignorance of the kind of calculus with which we have to deal we might perchance be adding to its size instead of dissolving it. The lithoprione by making us acquainted with the intimate nature of the stone would enable us to choose with certainty the reagent capable of destroying it." It seems impossible to overlook the similarity of ideas here with those of Arnott already quoted. Even the language rendered back into English is almost the same. In his "Exposé," too, M. Leroy tells us that he had suffered his mind to go astray after the idea of constructing a pouch in which the stone might be inclosed, and then dealt with by means of appropriate chemical reagents. M. Nicod tells us that Ducamp got entangled with a net or pocket with which he proposed to surround the stone and keep the fragments of it together after having broken it down. M. James Leroy it seems did so likewise: "To one of the modifications of the lithoprione," he says (Exposé, p. 168), "you may adapt a net to retain the fragments," &c. Now, though we may expect that ingenious men in pursuit of the same object will exhibit some similarity in their means of attaining it, still they will differ from one another in some particulars, their contrivances will not be identical. When they are, we must presume that they come from a common source.

The instruments of M. Amussat and M. Leroy were as it happened accidentally laid together before the Royal Academy of Medicine in the month of July, 1822. They were of the construction already described; that of Amussat being Hunter's forceps with the blades separated, and acting by crushing or breaking the stone in pieces; that of M. Leroy being a cage of four watch springs for fixing the stone, which was then to be reduced into fragments by being repeatedly pierced with a trephine or borer, moved with a bow in the manner of an ordinary drill. The Academy immediately appointed a committee to report on the capabilities of the two instruments now laid before it, and experiments on the dead body were forthwith ordered and instituted in the presence of the committee by the two inventors. One of the hardest kinds of calculi of the oxalate of lime was chosen for the proof. Unfortunately M. Amussat's instrument ("*ingénieusement conçu, mais grossièrement exécuté*," says Leroy, Hist. de la Lithotritie, p. 21), broke without being able to bite the stone. M. Leroy had rather better luck; the stone was seized with his lithoprione and pierced, turned and pierced again and again, so that poor M. Amussat was thrown completely into the shade, and he appears to have retired from the field discomfited with his ill-made, but really more available, instrument.

M. Leroy, though he carried off the palm on this occasion, does not seem to have been altogether satisfied with his lithoprione. He went to work again, and in the middle of April, 1823, presented to the Royal Academy of Surgery a second instrument, greatly simplified, and infinitely more facile of manipulation than the former one. With this instrument the stone was readily seized; it had but to be touched, in fact, and the blades expanded over it in order to be embraced. Instead of proposing to reduce the stone into pieces by repeated perforations, very tedious at all times and sometimes impossible, M. Leroy had now also contrived a slender rats-tail file, mounted upon a spring which gave it a tendency to diverge from the straight line. This file introduced into the perforation first made and worked by the bow, soon scooped out the whole interior of the stone and reduced it to a mere shell, which by and by could be crushed into fragments by the pressure of the blades of the forceps—a very happy idea. M. Leroy seems, however, to have found no opportunity of trying this instrument on the living body, and as this essential element was wanting, it seems to have attracted much less attention than it deserved. This second instrument of M. Leroy, however, united every element of excellence : it was as perfect as an instrument acting on the principle of perforation could be.

It was not until the year 1823 that we hear anything of M. Civiale in connexion with the subject of lithotrity. In this year he published a small work entitled : "*Nouvelles considérations sur la rétention d'urine ; suivies d'un Traité sur les calculs urinaires, sur la manière d'en connaitre la nature dans l'intérieur de la Vessie, et la possibilité d'en opérer la destruction sans l'opération de la Taille.*" The part of the work on strictures of the urethra is a mere epitome of Ducamp, and therefore of Arnott, or of Arnott and therefore of Ducamp. Had Ducamp and Civiale been friends (and we do not know that they were not), we should almost imagine that the former had said to the latter, *regardez un peu mon ami*, here is an ingenious enough little book, of one portion of which I have made such good use that my fortune is secure; there is another portion which I have not touched, but which well worked out may possibly do as much for you. M. Civiale, in the introduction to the Treatise on Calculus, tells us that having witnessed a most cruel operation for stone in the year 1817, which ended fatally, he began to think of finding some means of avoiding the necessity for having recourse to it altogether. He therefore informed himself of what had been done on this subject, ("Je pris connaissance des travaux qui avoient été fait à ce sujet,") and, satisfied that the notion which he had conceived was quite new, and that by its means it would at all events be possible to ascertain the composition of calculi in the bladder, he consigned the results of his meditations and of his experiments in a short memoir addressed to the Society of the Old Faculty of Medicine of Paris in the year 1818. This memoir in the work before us is alluded to under the title, "*Quelques Détails sur un Lithontriptique*," by which M. Civiale says he meant an instrument for grinding stone in the bladder; but on referring to the notification of its reception at the society we observe it designated *an instrument for the operation of lithotomy* (Bullet. de la Société de la Faculté de Médecine, Aout 1818). Grave doubts have from the very first discussion of the subject of lithotrity been raised as to the truth of these early pretensions of M. Civiale. The authenticity of the document which existed in the hands of Baron Percy in 1824, and which was shown as that originally addressed to the faculty of Medicine, has been formally called in question, and as no satisfactory answer has ever been rendered by M. Civiale to this arraignment, and as more than one report of the Royal Academy of Sciences delivered when there had been ample time to sift the subject to the bottom, has put him altogether aside as the *inventor* of lithotrity, we must conclude that his pretensions are without foundation. We have only to do with M. Civiale, therefore, from the year 1823. Let us examine his method of removing stone from the bladder without cutting. It is proper to begin, he says, by dilating the urethra, and he refers to the Arabian method of treatment, and immediately afterwards to Cooper's case of extraction of calculi through the urethra, " Un chirurgien très distingué (Astley Cooper) a tout récemment fait choix, m'a-t-on assuré, de cette méthode pour extraire de la vessie les calculs qui ne sont past rop volumineux." To effect the dilatation bougies are objectionable; what he employs is a piece of the intestine of a cat, which is introduced upon a bougie or sound, and this being withdrawn the gut, which is left behind, is then injected with water or air, till the dilatation deemed necessary is obtained. After this the *lithontriptor* is to be introduced. This is an instrument constructed of two hollow cylinders or canulæ, of such dimensions relatively that the one can readily be received within the other, and of a stylet which in its turn is received within the inner canula; connected with the inner canula are four branches, the elasticity of which causes them to separate, unless they be kept together by being drawn somewhat within the outer canula. The stylet is a very important part of this instrument, and has two principal objects, viz. to aid the elasticity of the branches and effect their separation, and to attack the stone when once it is seized. Now, on looking at the figure of M. Civiale, we see this essential difference between the drawing and the description, that in the former the blades of the forceps are *jointed,* whilst in the latter they are spoken of as possessed of *elasticity* to cause them to separate; they are jointed however; each consists of two pieces connected together by a simple hinge; and it was this structure that made the inferior cone of the perforator necessary; the blades of the forceps possessed of elasticity would have required nothing of the kind to make them expand; but as drawn in the figure, the blades have no elasticity, and could only have been opened by the backward pressure of a wedge or cone. In considering this figure and the description given of it we see positive evidence first of a want of mechanical skill, and then of harmony between them; the drawing and the printed account do not agree; the description looks like an after-thought. Such a machine was certainly unavailable; it never could have been used to seize and perforate a stone in the bladder; there is absolutely nothing for the operator to hold by, and the drill is to be worked by being turned between the fingers! But the *savoir faire* is of more avail in the world than the *faire savoir,* and if M. Civiale had little mechanical ingenuity, he seems to have had an ample endowment of worldly wisdom. Such as his miserable instrument was, it was presented along with a memoir entitled " Nouveau moyen de detruire la Pierre dans la Vessie sans l'opération de la Taille," to the Academy of Sciences in March 1824; and this illustrious body having appointed M. Chaussier and Baron Percy to report upon the memoir and instrument, Baron Percy drew up a document that at once arrested the attention not only of France but of Europe, and placed the obscure Civiale on a pinnacle from which he has continued to overlook not only all the men of his standing but almost every other, however eminent, in his own walk in the profession. To us, indeed, M. Civiale appears to be one of those favoured children of fortune for whom, to use a vulgar phrase, the bowls always run aright; one of those who without speed is nevertheless foremost in the race, who wins the victory or ere the battle be joined. M. Amussat charges a stone in a dead man's bladder, and his instrument fails him at the very onset, it goes to pieces, and he is disarmed and retires from the struggle. M. Leroy succeeds him, and makes a hole or two in the stone; but it is a tedious business; all present get tired, and the instrument is at length withdrawn, the stone being left behind but little the worse for the encounter. He perseveres, however; he sees the defects of his apparatus; he has the ingenuity necessary to remedy them, and is effectually implemented at length; but he finds no opportunity to try his fortune on the living body for something like a year, and when the opportunity does at last arrive, the circumstances are unfavorable and he fails. How different the course of the man on whose birth the genius of good luck has waited! He publishes a machine inferior in point of conception to every other already contrived, imperfect to such a degree that it never could have been used, and he has hardly proclaimed himself ready to take the field, when he finds not one but two, nay three occasions as "happy prologues to the swelling act" of his final greatness. His own instrument would not have served him indeed, but what signified this? M. Leroy had been sent before by Providence to provide him tools, and to work he went. Of course he succeeded—succeeded signally! In the first case which he encountered the patient was delivered of his stone in two sittings (Jan. 13th, 1824); in the second case (Feb. 4th) the stone was reduced to powder in four sittings; in the third case (March 4th) it was destroyed with the same ease and expedition. It was on the strength of these triumphs that Civiale came before the Royal Academy of Sciences, and it was with his mind's eye still dazzled with their splendour that Percy drew up the report to which allusion has been made, in which infinitely more than was his due is given to Civiale, and far less than belonged to him by indefeasible right is granted to M. Leroy. Civiale had indeed the good luck to find the opportunity of performing the operation on the living subject first, but he contributed nothing to the means by which he triumphed. His boasting in regard to lithotrity must be viewed as of a piece with that of the poor furnace-man, who lights the fire and turns on the steam, and then arrogates to himself the beautiful mechanism of the steam-engine and all the wonders it performs. The world is apt to say, the great merit of everything lies in its application. True, if this thing have been abandoned by its projector, and be rusting for want of use; but it is otherwise when the inventor is still occupied in perfecting his work, and is not only waiting but eagerly looking out for the opportunity to bring it into play himself. And this was precisely the case with M. Leroy and lithotrity; he has shown himself a good workman in numerous instances since, and lithotrity was altogether independent of the interference of M. Civiale; it would have been no less advanced had he never existed. M. Leroy's countrymen did him something like justice by and by, and, we think, all he has had is well deserved; for, in spite of our strong persuasion that the idea of removing the stone from the bladder, and even the first hints of the means of accomplishing this end, were not so original as he has by this time brought himself to believe, we feel it impossible not to admire M. Leroy for his mechanical ingenuity, and for the temper and forbearance he has shown towards M. Civiale, who certainly has exhibited none in regard to him.

Lithotrity, as we have followed it hitherto, had for its object the wearing down and comminution of the stone by perforations performed repeatedly and in different directions. But this is not the only mode of getting rid of calculi that has been imagined or that has been employed. Between the practice of extracting small stones from the bladder (1821), and the idea of breaking up such as were a little too large to be brought away, the distance is not great; and accordingly we have already seen that the maker of Cooper's forceps, Weiss, almost at the same time gave to the blades of this instrument such strength as he believed would enable them by their spring power to crush calculi into pieces. By and by (Feb. 1823), at the suggestion of that excellent surgeon, Mr. Thomas Davis, apparently, Weiss increased the strength of this forceps still farther; so far, indeed, that the action of a screw became necessary to open the blades. We have also seen that M. Amussat's instrument acted on the crushing principle, and that Elderton's, although not proposed, was applicable in the same direction. The idea and even the practice of breaking down the stone is, therefore, almost as old as that of wearing it down by perforations. Rodriguez, a surgeon of Malaga, delivered a patient of his stone among other means by striking or pounding it with a catheter every day or every other day (Alibert, in Journ. des Connais. Méd., tom. i.), and it is matter of tradition that the late distinguished surgeon, Mr. Thomas Blizard, when he met with a soft calculus, was in the habit of breaking it to pieces by means of a steel sound or catheter passed into the bladder.

Mr. Weiss, having made an instrument with a screw handle to *open* the blades, seems very shortly to have seen the vast advantage he would gain by using the screw to close them; and in the course of the same year he actually constructed an instrument, which may still be seen at his shop in the Strand, which differs in no essential particular from the screw lithotrite now in general use in this country, and also extensively known on the continent as Heurteloup's Percuteur. This last instrument, however, acts by the blow of a hammer, not, save adventitiously, by the pressure of a screw like Mr. Weiss's; but the two instruments are nevertheless essentially the same, the means only of procuring the power are different. M. Leroy seems also to have turned his attention to this mode of destroying the stone, and Professor Jacobson of Copenhagen at a later period contrived a very beautiful and very effective modification of the crushing lithotrite, which with a slight alteration from the hands of Dupuytren is probably to be reckoned among the most generally available instruments we possess.

We shall pursue this subject no farther. Our instruments are quite perfect, in so far as the end to be attained by their means is concerned; in the vast majority of instances no difficulty is now experienced in seizing and reducing to pieces even large calculi still contained within the cavity of the bladder. But has this object so eagerly pursued, so completely attained, answered the expectations that were even as dearly cherished of its being useful to humanity? We answer unhesitatingly

*No!* The value of lithotrity as a general means of treating stone in the bladder has been immensely overrated, and its indiscriminate application to all kinds of cases has cost many valuable lives. To such an extent has this already occurred, that it might be made a question whether M. Civiale's first successes ought not rather to be made subject of regret than of rejoicing, for successes in desperate operations are known to do vast mischief in the long run; one is saved, ten perish prematurely in consequence. It has hitherto, however, been a most difficult matter to get at the fact of the advantages or disadvantages of lithotrity. Operators generally have been somewhat chary of saying much upon the subject of the mortality. M. Civiale is in fact probably the only man who has had such ample personal experience of lithotrity as to be in a condition to speak from a large number of cases upon this point. But we regret to find that no credit whatsoever is due to the reports of M. Civiale. In his Traité de l'Affection calculeuse, p. 613, he speaks of the number of persons affected with calculus who had sought his assistance up to the year 1836. They amounted to 506. Of these 199 were either unfit subjects for lithotrity or were otherwise prevented from submitting to the operation. Supposing the whole of these to have been really unfit, this would be in the proportion of one in two and a half very nearly, to whom lithotrity held out no chance of relief, or who, were they subjected to the operation, would almost certainly lose their lives. We have, therefore, 307 subjects held favorable for the operation. Of these, says M. Civiale (Op. cit. p. 630), 296 were completely cured; 7 died; 3 were only partially relieved; and in 1 the issue is not known. But in the face of this very flattering statement let us turn to one or two of the public documents we possess and see how the conclusions in these accord with the numbers of M. Civiale. In a report presented by Messrs. Larrey and Boyer to the Royal Academy of Sciences in the month of April, 1831, upon the cases of calculus in the Hôpital Necker under Civiale, we find these respectable men challenging M. Civiale with dwelling upon his successful cases only, and making no mention of those who underwent lithotomy, lithotrity having been previously essayed in vain. "We find," say the reporters, "that twenty-four patients (not sixteen, as stated in the compte rendu,) had undergone the operation of lithotrity or lithotomy. Of these twenty-four, of whom *six* were cut [we presume after lithotrity had been tried in vain], *eleven* died more or less immediately after the operation." *Eleven* deaths in *twenty-four* cases, immediately after the operation! Verily there is little to boast of here, and we can already afford M. Civiale his seven deaths in 307 cases, and have four at our disposal to carry to the next account. Let us go on to public document the second. This is a report to the Royal Academy of Sciences presented by Messrs. Boyer, Double, and Larrey upon the operations performed at the Hôpital Necker during the years 1831 and 1832. "Fifty-three patients affected with calculus were received at the hospital. Of this number twenty-seven treated by lithotrity were discharged completely cured; sixteen having had various attempts at lithotrity made upon them, the operation was definitively found impossible or useless, or it proved fatal. Of these sixteen ten died and six remained unrelieved. Eight other patients were subjected to attempts at lithotrity and then to lithotomy; of these five died and three recovered." If we analyze this statement we find fifty-three receptions and twenty-seven recoveries; this is as nearly as possible one recovery in two cases by means of lithotrity, but as three recovered after the failure of lithotrity by means of lithotomy, we have three to add to the list of cures, which therefore amount to thirty in all. On the other side we find ten deaths as immediate consequences of lithotrity, and five more after lithotrity and lithotomy combined, that is fifteen deaths in all. Eight cases remain unrelieved, which must terminate fatally within a brief period, all the more quickly, for the sounding, &c. they doubtless underwent at the Hôpital Necker. *Fifteen* dead, *eight* unrelieved and expecting death, make twenty-three cases of non-success, to twenty-seven of success, by lithotrity. Surely neither is there aught to be proud of here; the cases in which no relief could be afforded were within *four* of being as numerous as those in which lithotrity was found of avail! And this is an operation that boasts of all but invariable success! three hundred and odd cures to seven untoward results!

Turning from public documents, let us see what some of M. Civiale's contemporaries have made out by an analysis of his cases, somewhat more rigorous than his own. M. Velpeau,* than whom there is no more honorable man or trustworthy writer in France, has given an analysis of five series of M. Civiale's cases, and we here append his table:

| Series. | No. of Cases. | Cured. | Dead. | Unrelieved, the stone remaining. | Otherwise Success in. | Otherwise Failure in. |
|---|---|---|---|---|---|---|
| 1st. | 83 | 41 | 39 | 3 | 41 | 42 |
| 2d. | 24 | 13 | 11 | 0 | 13 | 11 |
| 3d. | 53 | 30 | 15 | 8 | 30 | 23 |
| 4th. | 30 | 18 | 8 | 4 | 18 | 12 |
| 5th. | 16 | 6 | 7 | 3 | 6 | 10 |
| | 206 | 108 | 80 | 18 | 108 | 98 |

That is to say, of 206 patients operated on, 108, (a very little more than *one in two*) recover immediately; 80, or nearly *one in two and a half* die; and 18 retain the stone, and will be lost. *One hundred and eight cases cured, ninety-eight in which death* is immediately induced or may not be averted within a brief interval of time. This is a very different tale from the one told by M. Civiale himself; the reader may adopt the conclusions of whichsoever of the two statements he pleases. There can be no question as to the one that conveys the truth.

The experience of several other practitioners, excellent surgeons, with heads to plan and hands to execute this most delicate operation of surgery, but who have not addicted themselves exclusively to lithotrity, have not met with even the very moderate success of M. Civiale from its resources. M. Velpeau, however, has been more fortunate, and as his experience and candour are greatly to be relied on we shall give a summary† of twelve cases of calculus that were under his own immediate care, and in which he essayed lithotrity. In the first case, lithotrity had to be abandoned on account of the sufferings of the patient who remained unrelieved; in the second case the patient was cured; in the third he died; in the fourth, lithotrity had to be relinquished, after which,

* Médecine Opératoire, 2de Ed., t. iv. p. 649. Ibid. p. 653.

lithotomy was performed; in the fifth, the operation of lithotrity was also found impracticable, and lithotomy was had recourse to; in the sixth, the patient was cured; in the seventh, he was also cured; in the eighth, he died; in the ninth, he was cured; in the tenth, he died; in the eleventh, he was cured; in the twelfth, lithotrity had to be given up, and lithotomy substituted. Of the 12 cases, consequently,

5 were cured by lithotrity, (not one in two);
in 1 the operation was abandoned, and the patient remained unrelieved;
in 3 lithotrity was given up as impracticable, and the patients were cut, and recovered;
and 3 died of the operation.

5 in 12 is *one in two and a quarter*, nearly, in which success followed the operation of lithotrity.
4 in 12 is *one in three* in which lithotrity is unavailable, the operation cannot be performed.
3 in 12 is *one in four* in which a fatal result ensues.

The effect of the operation gone on with in the four cases in which it was abandoned may easily be conceived; it were not saying too much to maintain that in the hands of a man *committed* to the operation of grinding or crushing, four deaths more would have been added to the list of the mortality, when we should have had five recoveries counterbalanced by seven deaths. Other practitioners have, as we have hinted, had even less to boast of than M. Velpeau. And here be it observed that the deaths in all the preceding statements are the deaths that happen immediately upon the operation of lithotrity; there is no mention of those that occur at the distance of a few months, hardly of a few weeks, from the date of its performance. Were these taken into the account, the number of *cures* would be found wofully diminished, that of *deaths* frightfully increased. Our own knowledge and the published statements of more than one unimpeachable authority would lead us to maintain that the operation of lithotrity was even less fatal immediately than it is in brief prospective. In great numbers of instances the unfortunate patient who has undergone lithotrity *successfully*, i. e. who has got rid of his stone, and has not fallen an immediate victim to the means that have been used to deliver him, of course feels greatly relieved for a season; the stone is gone, the thorn in the living flesh is plucked out, and the patient rallies and again looks abroad upon the world with an eye of hope and gladness; but he is not quite well, more or less of irritability of bladder remains behind that soon renders the constant services of the medical attendant again necessary; this irritability increases; the patient begins to be tormented with ceaseless pain in the region of the bladder, which by and by extends up the loins and settles in the small of the back. The renal secretion has altered at an early period of these untoward symptoms; by and by it becomes like turbid whey, it has a faint sickly smell; it coagulates on the addition of nitric acid, and when exposed to heat; the patient loses strength rapidly; his stomach fails him; he becomes sick, and vomits; he begins to dose; frequently he has an epileptiform fit or two; then he falls into a state of coma, from which he never awakes, or he is seized with a more violent convulsion in which he expires. This picture is from the life; such are the symptoms which we have seen in more than one instance, occurring *within two years of successful litho-*

*trity.* Chronic disease has been excited in the bladder and urethra, and this by continuity of surface and of tissue creeps slowly upwards till the kidneys are attained, when death in the exhausted constitution in which such things take place is inevitable.

Mr. Fergusson (Edinb. Med. and Surg. Journal, Oct. 1838,) and Mr. Key (Guy's Hospital Reports, vol. ii. p. 1,) have each conferred a great boon upon true science by their observations upon lithotrity, and have effectually stripped the especial professors of this operation of the cloak of mingled pretension and mendacity with which they have robed themselves ever since they appeared upon the stage. Both Mr. Fergusson and Mr. Key have followed many patients upon whom lithotrity had been performed into their privacy, and have presented us with a sad picture of the life which these unfortunates generally lead, and of the miserable end which most of them meet at no long period after their deliverance from stone. Mr. Fergusson has recorded the particulars of seven cases in which lithotrity was performed, of which we wish our limits permitted us to present our readers with an abridgment seriatim, for their edification and enlightenment in regard to the value of this operation. All we can do is to lay before them an analysis of the results. In the 1st case the patient, though he was delivered from his stone, never recovered completely, and declared himself in worse plight than he was before he came under the hands of the lithotritist. In the 2d case the patient could not be delivered by the first series of operations; he had to retire with a fragment still in his bladder, which required a second series of sittings at the interval of more than a year, when he recovered. In the 3d, each operation was attended with such suffering that the patient, had he not been a man of great resolution, would have remained unrelieved. He recovered. In the 4th the patient nearly lost his life from the irritation induced by the attempt to operate by lithotrity. He was cut and recovered. In the 5th, lithotrity was found impracticable. The patient, cut twelve months afterwards, did well. In the 6th, the patient died four days after the operation of lithotrity. In the 7th, the patient could not endure lithotrity; he remained unrelieved, worse than before the operation. In these seven cases, therefore, we see but two *recoveries* from stone through the means of lithotrity. One man is delivered of his stone indeed, but he is left with a diseased bladder, which is, if possible, a worse evil than the stone. One is dismissed with his stone in fragments still contained in his bladder, and one dies immediately from the effects of the operation. That is to say, we have as good as three deaths in seven cases; for he who escapes from lithotrity with a diseased bladder dies, and he who is unrelieved of stone by one process, and is left beyond the pale of relief by any other perishes. Two are cut, lithotrity having failed, and recover. Two recoveries in seven cases, and each of these achieved at the cost of much suffering to the patient, and certainly with imminent peril to his life!

Besides these seven cases, Mr. Fergusson had cognizance of many others, in which lithotrity had been performed, but which were less immediately under his own eye than those the details of which he has given. Out of eighteen cases, however, in which he had known lithotrity to be performed, he informs us that *six were cured;* that *seven were not cured;* and that *five died.* Even in the number of reputed cures there is one case in which there are strong reasons for suspecting a *return* [quere, *a continuance*] *of the disease ;* there is a second, in which, though no stone can be felt, " the patient has suffered almost as much since he was operated on as he did previously to coming under the surgeon's care. Indeed," continues Mr. Fergusson, "*in two only of these* [*eighteen*] *cases can the operation be said to have been attended with that happy success which has been generally claimed for lithotrity.*"

Mr. Key, in his excellent paper, shows even as clearly and forcibly as Mr. Fergusson, that lithotrity is an operation that is neither so universally successful, nor so extensively applicable as might be inferred from the statements of its professors. In illustration of this position he gives the particulars of twelve cases, which we should gladly lay at length before our readers, but of which our space only permits us to subjoin an analysis :

12 cases, of whom

3 were cured by lithotrity, and

3, after vain attempts by this means, were cut and recovered.

6 died, of whom *one* with abscess in the prostate soon after the operation, *four* with protracted sufferings in consequence of fragments remaining in the bladder, and *one* with disease of the bladder, brought on or aggravated by the operation, but whether any fragments were left in the bladder or not was not ascertained. We have consequently *one case in four* cured by lithotrity, and *one case in four* cured by lithotomy, after lithotrity had failed ; finally, we have *one death in two*, of all the patients who were subjected to this means, these patients having all or with a single exception been under the hands of one of the most skilful lithotritists of the day ; a man who, to his literary and scientific knowledge, the result of a most liberal general and professional education, adds a rare mechanical genius, and a dexterity that was never surpassed. If these be the results in twelve cases where the Baron Heurteloup presided, and where Charles Aston Key lent his countenance and assistance, we own ourselves utterly at a loss to imagine what the amount of disaster must be where less of knowledge, less of skill, and less of conscientious feeling, have the sway.

The conclusions to be drawn from these cases and these views are obviously melancholy enough in so far as lithotrity is concerned. And yet, when we reflect dispassionately and as physiologists and practitioners upon the nature of the entire process in this operation, we see it impossible that the results could have been very different from what they are. Let us only consider the immediate consequences of the successful administration of lithotrity, the searching for and seizure of the stone, the necessary violence that accompanies the act of its comminution, and its condition with reference to the bladder after having been reduced to pieces, and we perceive that in the nature of things it can be no trifling operation, that on the contrary it must needs be one fraught with much danger to the patient. We know that the mere act of searching the bladder with a polished sound is often accompanied by a great amount of pain, and followed by what appears a singular degree of sympathetic disturbance ; we know that the attempt to seize and extract small stones in the bladder by the most delicate forceps has ended fatally; and how shall the necessarily large and complicated implements of lithotrity be introduced and brought into play within the bladder without producing a hundred times the amount of excitement and of mischief? This cannot be, and is not. And then, what shall we say in regard to the jarring and violence inseparable from the process of working a drill, or of turning a screw, or of giving the whole apparatus a smart blow with a hammer? What of a stone, which with a smooth surface was already such a source of suffering as to make the possessor weary of his life, and willing to take the chance of any odds against the solitary hope of obtaining relief, either roughened by repeated perforations, or reduced perchance into eight or ten angular and rugged fragments? All we can do is to admire the powers inherent in the delicate tissues that compose the excretory portion of the uropoietic system to withstand violence, and to repair themselves, bruised and maltreated as they necessarily must be, in such an operation as lithotrity performed by the most gentle hand.

The singular increase of irritation that takes place in consequence even of the *spontaneous* breaking up of calculi in the bladder, a phenomenon which sometimes occurs, and the danger to life that ensues thereon, is strikingly illustrated by the circumstances and the issue of a case which is related by Mr. Liston.* A medical man, who had laboured under symptoms of stone for a great many years, and who by sounding himself had ascertained the existence of a stone in his bladder ten years previously, was one day met by Mr. Liston in consultation. In three days after this Mr. Liston was summoned to this unfortunate gentleman in a moribund state, from inflammation of the whole urinary system, his urethra being at the same time blocked up by large fragments of stone. "It appeared," says Mr. Liston, "that on parting with me he had been summoned to an urgent case of labour. He ran quickly down a steep street, and at the bottom of it was seized with an urgent desire to make water, which he did in small quantity mixed with much blood. He passed some pieces of stone with sharp angles. He went on from bad to worse; he had retention, and the urethra was found much obstructed; suppression followed, and death terminated his sufferings in a few days. Many portions of the calculus were voided; much stone with the nucleus occupied the bladder and urinary passage. The kidneys were dark coloured, and one approached to a gangrenous state."

Now it is the business of lithotrity by a certain amount of mechanical violence, less or more, to accomplish such a disruption of a calculus as took place here spontaneously; and our amazement finally comes to be, how the operation should ever succeed, not that it should so often be found either impracticable, or if persevered in fatal. And this leads us immediately to consider the circumstances in which the operation is admissible, and those in which it is inadmissible. This point is soon discussed; the conclusion lies on the surface, and wants no farther fact or argument after what has been said to make it clear. Lithotrity is admissible and only admissible in cases in which the bladder is perfectly healthy, and in which the stone is small, of the size of a filbert, a shelled almond, or it may be a nutmeg at the utmost; under all other circumstances it ought to be held impracticable. In other words, lithotrity is admissible where it is estimated that the stone can at one sitting be seized and reduced to fragments of sufficient minuteness to be passed by

* Elements of Surgery, 2d ed. p. 632.

the urethra. No second, certainly no third operation ought ever to be contemplated. *If the patient who has had lithotrity performed upon him is not relieved at once, he is in imminent danger of losing his life.*

Lithotrity may now fairly be said to have been tried and found wanting as a general means of relief for stone. Restricted to the circumstances indicated above, it is a great addition to our chirurgical therapeia; applied indiscriminately, and as a substitute for lithotomy and all other means of treating stone in the bladder, it is a most fatal present made to humanity.

The next subjects we ought to examine are, first, the removal of the stone through an incision into the neck or fundus of the bladder—LITHOTOMY or CYSTOTOMY; and, second, its removal through an opening made into the urethra in perinæo and slow dilatation of the neck of the bladder—LITHECTASY, or CYSTECTASY: but we have already exceeded our prescribed limits in the length of this article; and important as the matter is, we feel that we must take another, and we trust we shall find an early opportunity, of discussing it by itself.

---

## Figures and Tables

**Figure f1:**